# What Zebrafish and Nanotechnology Can Offer for Cancer Treatments in the Age of Personalized Medicine

**DOI:** 10.3390/cancers14092238

**Published:** 2022-04-30

**Authors:** María Cascallar, Sandra Alijas, Alba Pensado-López, Abi Judit Vázquez-Ríos, Laura Sánchez, Roberto Piñeiro, María de la Fuente

**Affiliations:** 1 Nano-Oncology and Translational Therapeutics Group, Health Research Institute of Santiago de Compostela (IDIS), SERGAS, 15706 Santiago de Compostela, Spain; maria.cascallarc@gmail.com (M.C.); sandraalijasperez@gmail.com (S.A.); abi.judit.vr@gmail.com (A.J.V.-R.); 2 Centro de Investigación Biomédica en Red Cáncer (CIBERONC), 28029 Madrid, Spain; roberto.pineiro.cid@sergas.es; 3 Department of Zoology, Genetics and Physical Anthropology, Universidade de Santiago de Compostela, Campus de Lugo, 27002 Lugo, Spain; alba.pensado.lopez@rai.usc.es (A.P.-L.); lauraelena.sanchez@usc.es (L.S.); 4 Center for Research in Molecular Medicine & Chronic Diseases (CIMUS), Campus Vida, Universidade de Santiago de Compostela, 15782 Santiago de Compostela, Spain; 5 DIVERSA Technologies S.L., 15782 Santiago de Compostela, Spain; 6 Preclinical Animal Models Group, Health Research Institute of Santiago de Compostela (IDIS), 15706 Santiago de Compostela, Spain; 7 Roche-Chus Joint Unit, Translational Medical Oncology Group, Oncomet, Health Research Institute of Santiago de Compostela, Travesía da Choupana s/n, 15706 Santiago de Compostela, Spain

**Keywords:** zebrafish, nanomedicine, cancer, personalized medicine, drug screening, xenograft

## Abstract

**Simple Summary:**

Discovering new strategies for cancer treatment is critical, considering that each year millions of deaths are caused by this disease. In this sense, therapies based on nanomedicine are an innovative approach for cancer treatment, not only because they make it possible to perform targeted therapy, but also because they can improve patients’ quality of life. A key step to transfer new treatments from bench to beside is in vivo evaluation of a therapy, where zebrafish as a model organism has a fundamental role. Zebrafish has several benefits that make it ideal for studying the therapeutic capacity of novel nanotechnology-based anticancer therapies. In this review, we evaluate the potential of the nanomedicine and zebrafish synergy to achieve personalized treatments for cancer.

**Abstract:**

Cancer causes millions of deaths each year and thus urgently requires the development of new therapeutic strategies. Nanotechnology-based anticancer therapies are a promising approach, with several formulations already approved and in clinical use. The evaluation of these therapies requires efficient in vivo models to study their behavior and interaction with cancer cells, and to optimize their properties to ensure maximum efficacy and safety. In this way, zebrafish is an important candidate due to its high homology with the human genoma, its large offspring, and the ease in developing specific cancer models. The role of zebrafish as a model for anticancer therapy studies has been highly evidenced, allowing researchers not only to perform drug screenings but also to evaluate novel therapies such as immunotherapies and nanotherapies. Beyond that, zebrafish can be used as an “avatar” model for performing patient-derived xenografts for personalized medicine. These characteristics place zebrafish in an attractive position as a role model for evaluating novel therapies for cancer treatment, such as nanomedicine.

## 1. Emerging Cancer Therapeutics

Cancer is a major public health problem worldwide and the second-leading cause of death globally. Almost ten million people die from cancer every year, and this number is estimated to reach over 13 million in 2030 [1]. The most common causes of cancer death are lung, liver, and stomach cancers in men, and breast, lung, and colorectal cancers in women [2].

Cancer is a complex genetic disease that is caused by specific changes to genes in one cell or group of cells. These changes include sustaining proliferative signaling, evading growth suppressors, resisting cell death, enabling replicative immortality, inducing angiogenesis, and activating invasion and metastasis [3]. Metastases are the cause of the majority of human cancer deaths [4]. In the particular case of localized-stage colorectal cancer (CRC), the 5-year survival rate is around 90%, declining to 71% and 14% for patients diagnosed with regional and distant metastatic stages, respectively (American Cancer Society, Atlanta, GA, USA, 2017); in the case of pancreatic cancer, it falls from 37% to 3% in the distal metastatic setting [5]; and in non-small-cell lung carcinoma (NSCLC), from 24% to 6% in advanced stages of the disease when metastasis occurs [6]. 

The process of metastasis is defined by a cascade of complex events in which malignant cells detach from the primary tumor, invade through the basement membrane, and then migrate into the circulation, either via the blood or lymphatic vessels, to finally spread to distant sites to form metastases [7]. Although most early stage tumors can be surgically removed, there is growing evidence that dissemination could indeed happen at a very early stage in the carcinogenesis process [8], a fact that could explain why, in some tumor types such as pancreatic cancer, the 5-year survival even for localized disease is so poor.

It is generally accepted that the development of metastatic cancer implies cancer cells from the primary tumor alter several distinct features in order to succeed in this very complex process. Some of these modifications are (i) a change from an epithelial to a more mesenchymal phenotype, (ii) the acquisition of stem-cell properties and phenotypic plasticity, and (iii) a change in their metabolism in a way that promotes survival and metastatic outgrowth [9,10,11,12]. Conventional anticancer treatments mainly target the bulk tumor and often fail to eliminate the highly tumorigenic and chemo-resistant cell subpopulations.

NSCLC is a clear example of the results of these prevalent tumor treatments. Curative surgery is the standard of care for early-stage patients with good performance status; however, 35–50% of the resected patients relapse after an apparently successful surgical treatment [13]. For a long time, platinum-based doublets have been the standard first-line treatment option for unresectable advanced NSCLC [14]. Despite the survival improvement achieved with first-line chemotherapy, about 30% of patients do not obtain a tumor response. Moreover, patients who are initially sensitive to treatment acquire resistance and develop tumor progression after a median of about 5 months [15]. The current treatment strategy considers factors such as histology, clinical stage, age, performance status, comorbidities, the patient’s preferences, the molecular study, and an increasingly important focus on the immunological status. During the last years, a growing number of targetable major pathways have been identified, such as EGFR, PI3K/AKT/mTOR, RAS–MAPK, and NTRK/ROS1, leading to a new era of precision medicine [16].

Targeted therapy is the cornerstone of precision medicine, which seeks a molecular understanding of the disease to prevent, diagnose, and treat it. It can also be called personalized medicine, given that every patient receives the treatment that better fits their particular alterations in genes and proteins, providing them with significant responses and lesser toxicities compared with broad-spectrum cytotoxic therapy. The development of targeted therapies has resulted in substantial benefits in terms of survival and quality of life for cancer patients. Over the last twenty years, different drugs have been approved by the Food and Drug Administration (FDA) and the European Medicines Agency (EMA) for several tumor types, for example imatinib (gastrointestinal stromal tumors and dermatofibrosarcoma protuberans) [17] and cetuximab (colorectal cancer and head and neck cancer) [18], among others. 

On the other hand, immunotherapy has become the most revolutionary treatment in solid tumors [19]. The discovery of ligands and receptors regulating T cell activation, called immune checkpoints, has represented a major therapeutic breakthrough in the field. Immune checkpoint inhibitors (ICIs) are a group of antibodies designed to block specific targets present on tumor cells or lymphocyte surfaces (e.g., ipilimumab [20], the first approved anti-CTL4 antibody, and nivolumab [21,22,23,24] first approved anti-PD-1 antibody), consequently boosting the immune system to attack cancer. Immune checkpoints that target the programmed cell death-1 (PD-1), programmed cell death ligand-1 (PD-L1), and cytotoxic T-lymphocyte-associated antigen-4 (CTLA-4) have received approval across a wide range of cancer types, including lung cancer, melanoma, and head and neck, among others [16]. 

The current scenario for cancer research is wide and offers many possibilities for the constant improvement of therapies. In recent years, research into cancer medicine has taken remarkable steps towards more effective, precise, and less invasive cancer treatments. There is a plethora of newly proposed therapeutic options for cancer that are currently under investigation at different levels of maturity of the research stage, with some of them in clinical trials, such as oncolytic viruses [25], immune check-point antagonists [26], therapeutic cancer vaccines [27], natural antioxidants [28], hormone replacement therapy [29], exosome delivery platforms [30], aptamers [31], and thermal ablation and magnetic hyperthermia [32]. These strategies aim to provide the best personalized therapies for cancer patients and highlight the importance of combining multiple disciplines to achieve innovative approaches for the best outcome.

Other novel and promising therapeutic strategies that are already a reality in cancer treatment include antibody-drug-conjugates (such as ado-trastuzumab emtansine, approved in 2013 for treating HER2-positive metastatic breast cancer [33]); gene and cell therapy (such as tisagenlecleucel, approved by the FDA in August 2017 for certain pediatric and young adult patients with a form of acute lymphoblastic leukemia whose first-line drugs have failed [34]); and nanomedicine (for example, Doxil^®^, the first marketed PEGylated liposome loaded with the chemotherapeutic drug doxorubicin [35]). 

## 2. Nanomedicine and Cancer

Nanomedicine has been widely explored during the last decades. Different nanosystems composed of a variety of materials have been proposed for the management of several diseases, such as liposomes and other lipid-based and polymer-based nanoparticles, micelles, polyplexes, dendrimers, polymersomes, and drug/protein conjugates [36]. Nanotechnology offers many advantages in drug delivery, including (i) protection of drugs from premature degradation, (ii) increased solubility and stability in biological media, (iii) prevention of premature interactions of drugs with the biological environment, (iv) controlled pharmacokinetics and biodistribution, (v) improved delivery of therapeutics across biological barriers, and (vi) targeting of drugs to the diseased area [37,38]. Due to these properties and their ability to accommodate various types of drugs and biomolecules, with different physicochemical properties and activities, nanocarriers have emerged as attractive candidates for the development of personalized medicine [39,40]. Nanotechnology-based therapeutics are paving the way in the diagnosis, imaging, screening, and treatment of primary and metastatic tumors; however, translating such advances from the bench to the bedside has been severely hampered by challenges encountered in the areas of pharmacology, toxicology, immunology, large-scale manufacturing, and regulatory issues. The latest advances in nanomedicine and cancer have been extensively reviewed in recent works due to the high potential of this nano-based therapy to improve cancer patients’ quality of life [41,42,43]. A clear example is the work of Park et al., who reviewed how drug delivery systems progress over time, including cancer treatments such as Mylotarg^®^ and Doxil^®^ [44].

## 3. The Potential of Zebrafish for Preclinical Evaluation of Novel Cancer Therapeutics

Zebrafish (*Danio rerio*) is a vertebrate model species traditionally used for studying developmental biology and vertebrate genetics, and more recently, to model human diseases such as cancer, thus playing a key role in the discovery of new drugs for treating these illnesses [45,46,47]. Zebrafish characteristics define it as a model species between invertebrate models and murine models because it collects the vertebrate traits and allows large experiments [45,46]. One of the features that make zebrafish an appropriate human disease model is its homology with the human genome, around 70%, which increases to 82% in the case of human disease-related genes [48]. Furthermore, there are multiple advantages associated with the use of zebrafish, such as high fecundity and fertilization rate, producing a large offspring [49]. In addition, the external fertilization and optical transparency of embryos and larvae allow direct visualization of the overall development and enable the imaging of cells without the use of invasive techniques [50].

In terms of cancer research, aside from the robustness of zebrafish embryos to be easily manipulated, the adaptive immune system is not active until 2–4 weeks post-fertilization (wpf), and complete immunocompetence is only achieved at 4–6 wpf [51]. This feature, together with the previously mentioned transparency, enables the transplantation of fluorescent cancer cells (xenotransplantation or xenograft) and the visualization and tracking of their growth, biodistribution, metastasis, and neovascularization processes, as well as the evaluation of drug responses [50,52]. The main advantages and disadvantages of zebrafish as a model for human diseases are summarized in Table 1. 

The set of these characteristics have allowed researchers to develop several genetic and xenotransplantation zebrafish models and thus unravel the cellular, molecular, and physiological basis of different types of cancer, as well as drug response/resistance processes. Some relevant studies are reviewed in the following sections. 

### 3.1. Genetic Models

#### 3.1.1. Forward Genetics

**Figure 1:**
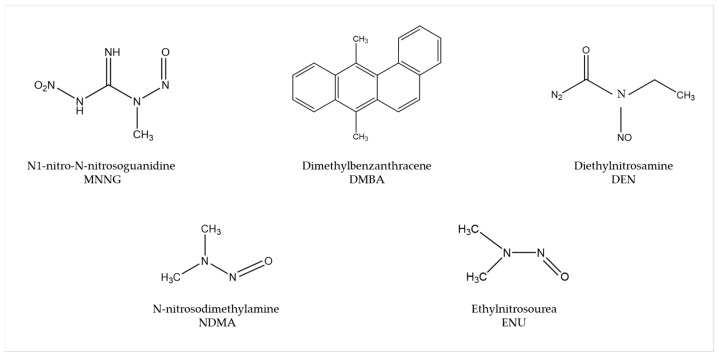
Most common carcinogenic substances used for tumor induction in zebrafish.

Several carcinogens are able to induce human-like tumors in different zebrafish organs (Figure 1) [53]. Thus, studies have been performed, allowing a better understanding of the carcinogenesis process, main target tissues, type of tumor, signaling pathways, and chemoprevention measures. For instance, exposure to N1-nitro-N-nitrosoguanidine (MNNG) in 86 h post-fertilization (hpf) embryos and 3 wpf fry (immersion), 72 hpf embryos (microinjection), and 6 wpf juveniles (diet) showed that embryos and fry are responsive to carcinogenic effects, whereas juveniles are remarkably resistant to neoplasia [54]. Embryos developed mainly hepatic and mesenchymal neoplasms, including chondroma, hemangioma, hemangiosarcoma, leiomyosarcoma, and rhabdomyosarcoma. The blood vessels and testis were the main target organs in fry, developing seminoma, hemangioma, hemangiosarcoma, and various other epithelial and mesenchymal neoplasms. Similarly, it has been shown that exposing zebrafish to Dimethylbenzanthracene (DMBA) at 3 wpf led, principally, to hepatic neoplasia in adults [55], with conservation of human transcriptome profiles, highlighting the potential of zebrafish for modeling human liver cancer [56]. Maid, a protein involved in cell proliferation, is abundantly expressed in the liver hepatocytes’ cytoplasm of zebrafish; its role as a regulator of hepatocarcinogenesis was explored by treating adult zebrafish with Diethylnitrosamine (DEN) for 8 weeks. After treatment, these fish presented distended abdomens, extremely swollen livers, and different types of liver tumors. However, Maid appeared to translocate from the cytoplasm to the hepatocyte nucleus, presumably to participate in growth-inhibitory signaling and display its tumor-suppressor activity [57]. It has been stated that polyploidy in lower vertebrates decreases the probability of inactivation of all alleles of tumor suppressor genes, so the incidence of tumors might be lower [58]. In this regard, the relationship between polyploidy and tumor formation has been investigated through N-nitrosodimethylamine (NDMA)-induced hepatocarcinogenesis [59]. Diploid and triploid 6 wpf zebrafish exposed to this chemical for 8 weeks developed hepatocellular adenomas and trabecular hepatocellular carcinomas after 24 weeks from the beginning of the treatment, although cholangiolar tumors were not detected in triploid fish until 36 weeks, serving as evidence that polyploidy is a protective factor in pathogenesis of this type of tumor, probably indicating a lower probability for putative tumor suppressor genes to be inactivated in polyploid cholangiolar cells. 

The mutagen ethylnitrosourea (ENU) has been used to generate point mutations, leading to the identification of several mutant zebrafish lines with an increased incidence of spontaneous neoplasia or higher sensitivity to chemical exposure [53]. For instance, Basten et al., in an attempt to study ciliary motility defects in the lrrc50 mutant zebrafish line, unexpectedly found development of seminomas in 2-year-old adults, with a penetrance of >90%. This observation allowed establishment of a correlation between the gen and such testicular germ cell tumors (GCTs) and proposes lrrc50 as a novel tumor suppressor [60]. Similarly, Neumann et al., while screening for cancer susceptibility genes, isolated a zebrafish mutant line with highly penetrant, heritable testicular GCTs in which testicular tumors spontaneously developed. Indeed, DMBA or MNNG exposure resulted in enhanced germ cell tumorigenesis [61]. 

#### 3.1.2. Transgenic Zebrafish Lines

Several zebrafish cell and tissue-specific reporter lines have been developed over the last years to improve the comprehension and characterization of different cancer traits, such as tumor cell growth, migration, invasion, angiogenesis, drug responses, or interactions with immune cells. Some examples are Tg(mpx:GFP) and Tg(mpeg1:eGFP) [62,63], which fluorescently label neutrophils and macrophages, respectively, or Tg(fli1:eGFP) [64], which labels the vasculature. Furthermore, human or murine oncogene transgenic expression in zebrafish has also helped to understand their role in tumor development; for example, Tg(ptf1a:eGFP-KRAS^G12V^) in pancreatic cancer [65] and Tg(mitfa:HRAS^G12V^; mitfa:GFP) or Tg(mitfa:BRAF^V600E^); tp53^−/−^ for melanoma [66,67]. The binary transgenic system Gal4/UAS has also been extensively used. Gal4 is a transcriptional activator that, when expressed under the control specific tissue-specific promoters, binds to UAS enhancer sequences in the DNA, recruiting transcription machinery to induce gene expression, so genes under the control of UAS sites are expressed when Gal4 is present [68]. With this methodology, authors have shown, for instance, that crossing Gal4-expressing lines with Tg(UAS:HRAS^G12V^) transgenic line resulted in the development of different types of tumors, such as leukemia, glioma, or chordoma [69,70,71]. As transgenic fish with overexpression of some oncogenes might not survive to adulthood, transgenic inducible lines can also be generated, for instance, TetOn system-based transgenic lines, in which the oncogene expression is induced by doxycycline. Doxycycline-inducible expression of oncogenic KRAS in brain cells under the control of the krt5 and gfap gene promoters using the TetOn system (Tg(TRE:mCherry-KRAS^G12V^; krt5/gfap:rtTa)) led to the development of malignant tumors in the cranial cavity and parenchymal brain tumors, respectively [72].

#### 3.1.3. Reverse Genetics

Morpholinos (MO) are commonly used in zebrafish to achieve transient expression silencing without modifying the genome sequence [73] and thus to determine certain cancer invading mechanisms, such as angiogenesis. For instance, Jacob et al. reported that Plexin-A1 (PlexA1) could be a potential prognostic marker for glioma patients’ survival, as quantitative analysis correlates tumor grade and the level of PlexA1 expression in brain blood vessels [74]. They knocked down PlexA1 in Tg(kdrl:eGFP) zebrafish and observed a significant number of abnormal angiogenic sprouts in intersegmental vessels (ISVs) at 28 hpf, confirming the relevance of PlexA1 in blood vessel development. Royet et al. observed that high expression of Ephrin-B3 in human glioblastoma biopsies promotes tumor growth and angiogenesis by inhibition of EphA4-induced apoptosis [75]. They knocked down Ephrin-B3 in Tg(fli:EGFP) embryos and observed an impaired ISVs formation associated with an increase in apoptosis. Co-silencing of EphA4 resulted in the rescue of the angiogenic defects, suggesting that enhancing EphA4-induced cell death could be envisaged as a relevant strategy to slow glioblastoma (GBM) growth. 

In order to generate stable mutant models, Targeted Induced Local Lesions in Genomes (TILLING), based on the exposure to ENU and further sequencing [76], has been extensively used. In this sense, mutations in tumor suppressor genes, such as tp53, pten, and apc, have been identified in ENU mutagenesis libraries, and fish were found to develop malignant peripheral nerve sheath tumors (MPNSTs), ocular hemangiosarcomas, and intestinal adenomas, hepatomas, and pancreatic adenomas, respectively [77,78,79,80]. Interestingly, ENU homozygous brca2 mutants were shown to be unable to develop ovaries during sexual differentiation, developing as infertile males that were prone to develop testicular neoplasias during adulthood [81]. By combining the use of vhl zebrafish ENU heterozygous mutants and the exposure to DMBA, Santhakumar et al. established the von Hippel-Lindau protein (pVHL) as a genuine tumor suppressor in zebrafish, due to the increase in the occurrence of hepatic and intestinal tumors in mutants [82]. Although TILLING has proven to be useful to correlate genotypes with phenotypes, the difficulty involved in the screening process, together with the possibility of having further mutations than the one desired, led researchers to introduce other methodologies, such as nuclease-based techniques, which include Zinc Finger Nucleases (ZFNs) and Transcription Activator-Like Effector Nucleases (TALENs).

ZFNs were used to generate tet2 mutants, which developed progressive clonal myelodysplasia, culminating in myelodysplastic syndrome, with dysplasia of myeloid progenitor cells and abnormal circulating erythrocytes [83]. As it recapitulates human TET2 loss-of-function phenotypes, this model was proposed as a platform for small-molecule screenings to identify compounds with specific activity against tet2 mutant cells. The function of the neurofibromin 1 (NF1) gene in brain tumorigenesis was explored by Shin et al. through the generation of stable mutant lines for the zebrafish orthologs (nf1a and nf1b) by ENU and ZFNs [84]. nf1a+/−; nf1b−/− mutants in a p53 mutant background presented an increased penetrance of high-grade gliomas MPNSTs as well as hyperactivation of ERK and mTOR pathways, consistent with mouse and human NF1-derived MPNSTs and gliomas. Similarly, bi-allelic cdkn2a/b and rb1 mutants generated by TALENs developed MPNSTs and medulloblastoma-like primitive neuroectodermal tumors, respectively, in a p53 mutant background [85]. Nevertheless, the design of nuclease-based systems is challenging, and there is still a high rate of off-targets. Thus, the introduction of the CRISPR/Cas9 system has allowed the optimization of the genome editing protocols and the improvement of the efficiency and accuracy of zebrafish lines. 

For instance, p53/nf1-deficient fish were used by Oppel et al. to knock out by CRISPR/cas9 the atrx gene, a known tumor suppressor in gliomas or sarcomas, confirming that its loss facilitates the development of various malignancies, together with the downregulation of telomerase, which causes the alternative lengthening of telomeres [86]. Loss-of-function mutations in SUZ12, a subunit of the Polycomb repressive complex 2, have been identified in a variety of tumors, including MPNSTs. The knockout of suz12a and suz12b in a p53/nf1-deficient model significantly accelerated the onset and the penetrance of MPNSTs, and additional types of tumors were detected, including leukemia with histological characteristics of lymphoid malignancies, soft tissue sarcoma, and pancreatic adenocarcinoma [87].

**Figure 2:**
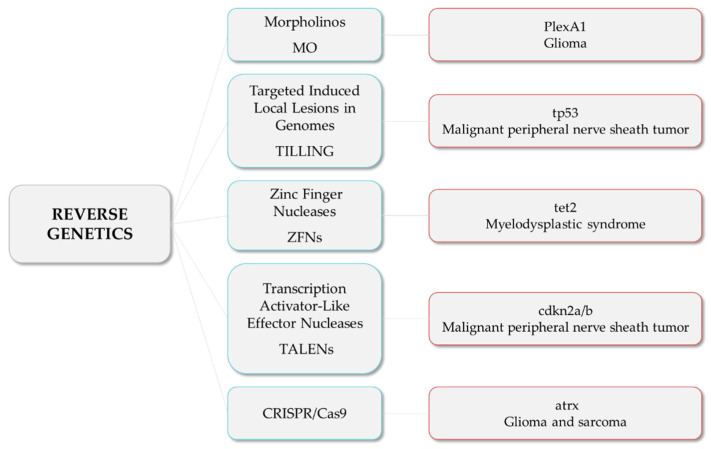
Reverse genetics strategies (in blue) and their respective examples of altered genes and the associated tumor types.

These are examples of studies in which researchers developed zebrafish models harboring mutations in tumor suppressor genes and novel candidate genes, among others, to investigate their roles and unravel the relationship among mutations and the tumorigenesis and progression of different types of cancer (Figure 2).

### 3.2. Transplantation Models

Transplantation in zebrafish is based on the injection of fluorescently labeled cancer cells into zebrafish embryos. The main transplantation techniques include allotransplantation and xenotransplantation, and both can be orthotopic or heterotopic, depending on whether the cells are injected in an equivalent anatomical site to tumor origin or into a different anatomical site, respectively. 

**Figure 3:**
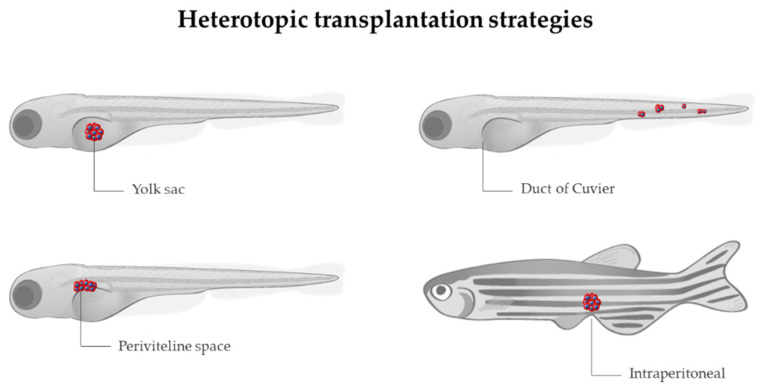
Sites for heterotopic transplantation of tumor cells (in red) in zebrafish. Modified from Servier Medical Art (https://smart.servier.com; accessed on 3 March 2022), licensed by a Creative Commons Attribution 3.0 Unported License, and Lizzy Griffiths.

Allograft consists of tumor cells being transferred from one individual to another of the same species [88], while xenograft is based on the injection of labeled human, murine, or patient-derived cancer cells into zebrafish, to track their survival, engraftment, growth, behavior, and interaction with the microenvironment [89]. The common sites for heterotopic transplantation are (Figure 3): (a) yolk sac, to track survival and proliferation [90]; (b) duct of Cuvier, to observe migration, invasion, and mesenchymal-epithelial transition (MET) [91]; (c) perivitelline space, to investigate mainly neovascularization [89]; intraperitoneal cavity, when adult individuals are used. The majority of transplantation assays are performed at 2 days post-fertilization (dpf), taking advantage of the transparency of the embryos and the lack of adaptive immunity, although several experiments have been carried out using adults. In order to avoid immune rejection in adults, transplantations require immunosuppression with sublethal γ-irradiation or dexamethasone [92,93] or the use of immunocompromised lines, such as the Rag2, lacking mature T-cells and having a reduced number of B cells, or the compound mutant prkdc−/−, il2rga−/−, lacking T, B, and natural killer (NK) cells [94,95]. In the particular case of allografts, engraftment can also be achieved without the need for immunosuppression by the transplantation from a donor fish to a genetically identical recipient (syngeneic or clonal models) [96], serving as a model for long-term engraftment and self-renewal potential [97,98,99]. An interesting approach combining different strategies allowed Ignatius et al. to confirm the role of tp53 in the development of a wide spectrum of tumors [100]. By using TALENs, they created tp53 mutants in which MPNSTs, angiosarcoma, germ cell tumors, and leukemia spontaneously developed during adulthood, and such tumor cells were transplantable to syngeneic fish, so engraftment of fluorescent-labeled tumors could be dynamically visualized over time. Additionally, White et al. proposed a mutant transparent recipient, known as *casper* zebrafish (roy−/−; nacre−/−), as a platform to study cancer cell engraftment, proliferation, and distant metastases in vivo [101]. Nevertheless, the prompt recovery from chemical immune ablation, the vulnerability of mutant immunocompromised fish, and the limited number of syngeneic zebrafish lines made embryo xenograft the most cost-effective technique, together with the higher number of individuals used, which increased statistical power and the reduced ethical issues in comparison with adults.

The first xenograft assay was performed by injecting melanoma cells in zebrafish blastula, which showed the ability of melanoma cells to survive, proliferate, and specifically migrate to the skin, highlighting the validity of the zebrafish for cancer research [102]. Since then, this technique has been improved and extended for studying several cancer features, including not only survival, proliferation, or migration, but also the ability to invade, form new blood vessels (angiogenesis), metastasize, and respond or resist different treatments. Additionally, researchers have made efforts to mimic human tumor conditions and microenvironments as much as possible, as reviewed by Cabezas-Sáinz et al. [103]. For instance, the use of transgenic zebrafish lines, such as the previously mentioned ones, labeling immune cells or vasculature, has led several researchers to better understand the interaction among tumor cells and macrophages or neutrophils, and their involvement in tumor growth, vascularization, invasion, and metastasis [104,105,106,107]. In this line, Allen et al. recently presented a new model for tumor cell extravasation of both individual and multicellular circulating tumor cells, known as angiopellosis, and their ability to form tumors at distant sites [108]. 

With the aim of recapitulating, not only the cellular but also the non-cellular environment provided by the specific site and/or organ orthotopic xenografts have been developed, mainly with brain tumor cells. A pioneering study was performed by Lal et al., in which GBM cells behaved differently when injected into the yolk sac or in the brain. While cells in the yolk were unable to proliferate or invade, cells injected orthotopically showed the ability to invade the brain and disperse along the vessels [109]. By combining MOs, orthotopic xenograft, and 4D individual tracking technology, Gamble et al. showed that laminin subunit alpha 5, an important component of blood vessels, increases the attachment of GBM cells to blood vessels, suppressing tumor invasion but promoting tumor formation [110]. Additionally, orthotopic brain xenografts have proven to be unique models to study the ability of different drugs to penetrate the blood–brain barrier [111]. Retinoblastoma has also been studied by orthotopic xenografts. The inhibition of Nodal using short hairpin (shRNA) reduced the ability of retinoblastoma cells to disseminate outside the eye, highlighting the importance of Nodal in promoting growth, proliferation, and invasion [112].

Although the above-mentioned techniques have helped to improve the knowledge of several cancer processes, tumors present high interindividual heterogeneity. In addition, established cancer cell lines often differ significantly from patients’ tumor cells. Thus, to preserve the patients’ tumor biological and genetic profile and improve the accuracy of tumor drug-response studies, zebrafish patient-derived xenografts (zPDXs) have arisen as a potential solution [106]. zPDXs are established from tumor cells or masses isolated from patients during biopsy or excision, which are subsequently hetero- or orthotopically implanted into zebrafish. The pioneers of this technique were Marques et al., who observed cell invasion and metastasis formation after injection of colon, pancreas, and stomach primary tumor samples into the yolk sac [113]. Since then, the survival, proliferation, angiogenesis, or invasion ability of different patient-derived tumor cells have been studied in zebrafish models, from pancreatic, colon, gastric, head and neck, or pituitary cancer, to abdominal liposarcoma or T-cell acute lymphoblastic leukemia [114,115,116,117,118]. Furthermore, in order to improve patients’ treatments, zPDXs have served as a platform to develop drug response/resistance assays and thus move towards personalized medicine. In this sense, several strategies are reviewed below.

## 4. Zebrafish as a Platform for Drug Screening

Zebrafish are used as a screening platform to adjust drug concentrations, to improve combinatorial treatments for a less toxic effect on the patient, or to overcome resistances, as well as a tool to study the mechanism of action of drugs in the organism and to alter the function of a biological pathway without previously knowing the components. Small molecule screening in zebrafish started in 2000 with a work by Peterson et al., who tested the effect of a variety of molecules in the development of vertebrate animals to understand how these molecules can be used to determine the timing of critical developmental events [119]. 

In the context of cancer, zebrafish xenotransplants have been useful as in vivo preclinical tools for drug testing. This approach has been validated by different works, showing its complementarity with other in vivo models such as the mouse [120]. However, xenotransplantation of human cancer cells into the zebrafish is not without difficulties. For instance, the normal growth of human cells is at 37 °C, and the temperature of development of the zebrafish is 28 °C. To overcome this issue, the field has established 31–34 °C as a consensus temperature for xenograft assays. However, these temperatures could cast some doubts about the efficiency of xenograft models for drug screening and the subsequent translation to the patient. In this sense, Cabezas-Sáinz et al. demonstrated that zebrafish larvae can live until 36 °C, allowing them to test drugs in a cancer model with characteristics close to humans [121]. In addition, Cornet et al. developed the ZeOnco Test, an optimized and standardized (regarding cell labeling, injection site, image acquisition, etc.) xenograft assay, aiming at reducing attrition rate [122,123]. In the same way, xenografted Tg(fli1:EGFP) transgenic models with human lung cancer cell lines were used to compare the effects of different known drugs, promoting these models as a real-time drug screening platform for clinical lung cancer patients [124]. 

Zebrafish have been used for the development of combined treatment approaches to improve treatment efficacy. An example of this goal was the investigation of Precazzini et al., where the melanoma kita:ras transgenic zebrafish line was used to test the antifungal Clotrimazol in combination with antitumoral drugs, showing a synergistic anti-melanoma effect with limited toxicity [125]. In addition, other authors used the *casper* transgenic line to evaluate individually and in combination the antitumor activity of chemotherapy drugs used in the clinic [126].

In addition to pharmacological cancer treatments, there are other treatment options, such as radiotherapy, based on the use of ionizing radiation. An example is the work by Costa et al., who combined ionizing radiation and chemotherapy in colorectal cancer tumors xenografted into the *nacre* (*casper* and [Tg(fli1:EGFP)]) zebrafish line and observed that the responses achieved in the zebrafish matched the clinical responses of patients [127]. 

Transgenic Tg(fli1:EGFP) zebrafish with fluorescent vasculature has been used in a wide range of screenings in studies related to the angiogenesis process, such as new synthetic compounds [128,129], analogous molecules for drugs in use [130,131], natural compounds used in traditional medicine [132,133], and natural analogous compounds [134]. The combination between fluorescent vasculature and xenograft transplantation offers a potent cancer research tool to study the action of compounds in vivo, in which to test the potential of natural products in anticancer therapy [135,136], modification of natural compounds [137], and new chemical compound structures that are already utilized in the clinic [138]. Lin et al. combined the fluorescent vasculature of zebrafish with other genetic modifications and cancer cell xenotransplantation to screen and identify new anticancer molecules [139].

Even so, wild-type zebrafish is also used for screening of new molecules obtained from marine organisms [140,141,142] as well as the study of the potential of some fungicides against cancer cells [143]. 

Furthermore, other specific transgenic zebrafish were used in the study of different tumor drugs. For instance, the vhl^hu2117^ mutant transgenic zebrafish, which shows an excess of vascularization, was used to evaluate the antiangiogenic effect of the compound largazole [144]. In addition, a transgenic zebrafish for liver cancer overexpressing the oncogene KRAS was used to study the effects of environmental toxicants on tumor development and inflammatory response [145].

**Figure 4:**
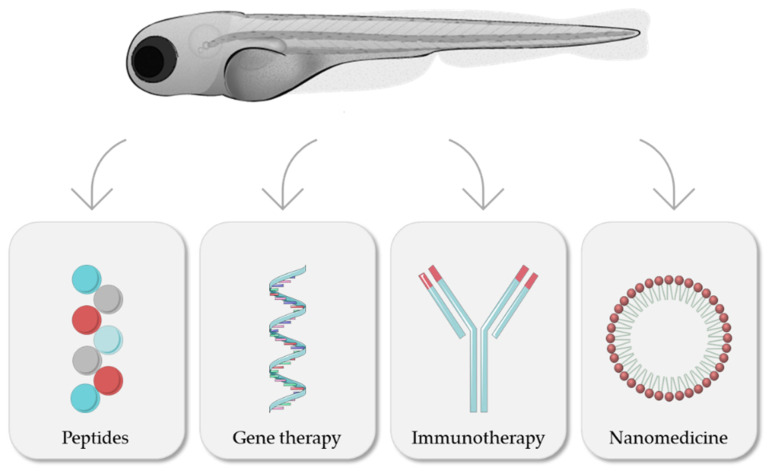
Zebrafish as a model for evaluation of different cancer treatments. Modified from Servier Medical Art (https://smart.servier.com; accessed on 3 March 2022), licensed by a Creative Commons Attribution 3.0 Unported License, and Lizzy Griffiths.

In the following sections, and as summarized in Figure 4, different therapeutic approaches for cancer treatment evaluated with the zebrafish model will be discussed.

### 4.1. Peptides 

Among the different types of biomolecules, zebrafish have proved their potential for evaluating the activity of novel peptide therapies. Cancer peptide-based therapy might play a role in the treatment of patients, and peptides can be obtained from different sources, such as natural organisms, peptide libraries, and de novo synthesis [146,147]. Some peptides produced by bacteria are also used to treat some types of cancer due to their antitumor effect. A shining example is the microcin E492, which is a peptide produced by the bacteria *Klebsiella pneumoniae*, which has shown antineoplasic properties in zebrafish embryos xenografted with colorectal cancer cells [148]. Another example, provided by Hsieh et al., studied the effect of TAT-NLS-BLBD-6, a synthesized peptide able to suppress breast cancer growth. This in vivo assay was carried out through a co-microinjection of peptide and labelled breast cells into the zebrafish yolk [149]. Furthermore, the evaluation of anticancer peptides in zebrafish embryos can be carried out by xenotransplantation of treated cells. An example of this assay was performed with NuBCP-9, a growth factor Nur77 derived peptide, which demonstrated apoptotic effect in paclitaxel-resistant lung tumor cells [150]. 

### 4.2. Gene Therapies 

Zebrafish have also been used in the research of gene therapies based on the introduction of exogenous genomic materials on the organism to study or silence the expression of genes. A significant example is the research performed by Cordeiro et al., who used a specific ssDNA in the fli-EGFP transgenic zebrafish to measure the capacity of this material to reduce the GFP signal; the reduction of the EGFP emission indicates the downregulation of EGFP expression [151].

Transgenic zebrafish line Tg(Kdrl:eGFP)s843 has been used as an in vivo model to study the antiangiogenic effects of miRNA-based therapies. This assay was carried out with xenografts based on miRNA transfected prostate cancer cells, which allowed the evaluation of new vessel formation [152]. In the same vein, Kiener et al. studied the antitumor effect of a miRNA in the same type of cancer by microinjecting transgenic Tg(mpo:GFP)i114 zebrafish with miRNA transfected cells, proving the reduction of the tumor due to the miRNA effect [153]. 

### 4.3. Immunotherapeutics: Monoclonal Antibodies and CAR-T

Monoclonal antibodies can target cancer cells by binding to their specific surface antigens [154]. Zebrafish embryos play a meaningful role in the study of the anti-cancer efficacy of monoclonal antibodies, their toxicity, and the comparison between different therapies.

Zebrafish were used to study cetuximab, a monoclonal antibody targeting the epidermal growth factor receptor (EGFR), for the treatment of colorectal and head and neck cancer [154]. The response of cetuximab treatment was evaluated using colorectal cancer zebrafish patient-derived xenografts (zPDX), which included the drug in the injected cell suspension, and the results showed that the zebrafish model allows the detection of differential responses to the antibody according to the *KRAS* mutational status of the tumor [155].

Furthermore, the zebrafish transgenic line Tg(fli1:EGFP) is commonly used to study the antiangiogenic capacity of drugs [156,157,158], such as Bevacizumab, a humanized anti-vascular endothelial growth factor (VEGF) antibody for the treatment of some solid cancers (such as breast and lung cancer). Bevacizumab was studied in this zebrafish transgenic line to define its antiangiogenic effect and antitumor capacity, in contrast to toxicity assay, which was performed in wild-type embryos [154,157,158]. Another monoclonal antibody studied in zebrafish models is ramucirumab, used to treat lung, gastric, and colorectal cancer. Its toxicity was assessed in wild-type zebrafish embryos, and the antiangiogenic and anticancer capacity was tested in the Tg(fli1:EGFP) line, in the same way as Bevacizumab [154,156].

Moreover, chimeric antigen receptor T cell (CAR-T cell) therapy has achieved clinical success in specific tumor types, such as several types of leukemia [159,160]. Recent studies by Pascoal et al., for the first time, evaluated the capacity of CAR-T cells to kill cancer cells in vivo in zebrafish. To carry out this assay, labelled Nalm-6 leukemia cells and CAR-T cells were injected into zebrafish vasculature. The results show that zebrafish embryos are a potential model for in vivo studies of the efficacy of CAR-T cell therapy against cancer [161].

### 4.4. Nanomedicines 

#### 4.4.1. Toxicity 

Zebrafish embryos are currently used for nanomedicine toxicity testing due to advantages such as their high fertilization rate, as explained in Section 3. The most common method to perform toxicity assays is the incubation of nanomedicines into zebrafish medium, usually with dechorionated zebrafish. As well as the incubation, microinjection of test drugs into zebrafish circulation ensures that the concentration is absorbed by the embryos [49].

**Table 2:** Zebrafish-based toxicity studies of different nanoparticles for cancer therapies.

Nanoparticles	Conditions	Higher Mortality Rate	Morphological Effects	Ref.
AgNPs	3 hpf embryos 72 h incubation 28.5 °C	100% (3 μg/mL)	Yolk sac edema Tail malformation	[162]
AuNPs	3 hpf embryos 72 h incubation 28.5 °C	100% (300 mg/mL)	Yolk sac edema	[162]
MMDOX	4 dpf embryos 72 h incubation 28 ± 1 °C	100% (100 μg/mL)	Uninflated swim bladder Arched body Alteration of the spontaneous swimming activity	[163]
MSNs-FA	48 hpf embryos 72 h incubation 27 ± 1 °C	~30% (200 μg/mL)	Hatching rate	[164]

Several aspects of zebrafish embryos can be analyzed to determine the toxicity of a specific nanomedicine; some examples are compiled in Table 2. The correct hatching process, malformation appearance, the response of the immune system, and mortality are some of the guidelines to evaluate the toxicity effect of nanoparticles [165,166]. As a result, toxicity tests based on zebrafish have become an indispensable step to assess the effect of several therapies based on nanosystems, from metal-based nanoparticles to lipidic nanosystems. For instance, golden (AuNPs) and silver (AgNPs) nanoparticles for anticancer application were tested to evaluate their toxicity using zebrafish embryos. Mortality rate and morphological anomalies showed differences between nanoparticle types and concentration [162]. However, metal nanoparticles are not the only kind of nanomedicines evaluated by the zebrafish toxicity test. In fact, micelles loaded with doxorubicin hydrochloride (DOX-loaded mixed micelles (MMDOX)), commonly used to treat metastatic breast cancer, were tested by Calienni et al. in zebrafish embryos to rate their toxicity in vivo [163].

Another important example is the research of Wu et al., who used zebrafish embryos to evaluate the biosafety of mesoporous silica nanoparticles coated with folic acid (MSNs-FA) as carriers of therapeutic peptides, evaluating the embryo mortality and hatching [164].

Zebrafish have demonstrated their huge capacity to be a platform for testing different types of nanomaterials, not only for cancer treatment but also for other applications such as antibacterial and heart-associated disease treatment. The review of Jia et al. compiled information about different nanoparticles and their toxicity evaluation using zebrafish [167].

#### 4.4.2. Biodistribution and Average Life in Circulation

In vivo behavior, distribution along the body, and interaction with tumor cells are key qualities to develop new anticancer nanomedicines; therefore, analyzing these aspects is essential to achieve a translation to the clinic of nano-based therapies. Due to this, zebrafish embryos play an important role as a platform to evaluate these properties in vivo. 

Chang et al. performed an assay to evaluate differences in the distribution of polystyrene nanoparticles and glycol chitosan nanoparticles for cancer treatment along blood circulation. Adult wild-type zebrafish were retro-orbitally injected with nanoparticles to observe their capacity to circulate along the vasculature; this allowed the authors to predict in vivo nanoparticle behavior [168]. In the same way, Gundersen et al. also used wild-type zebrafish to evaluate the biodistribution of chlorpromazine-loaded PEGylated PLGA nanoparticles for leukemia treatment [169].

In another fashion, transgenic Tg(FLK-1: mCherry) zebrafish embryos, in which endothelial cell membranes are fluorescently labeled, were used to evaluate the distribution of nanoparticles throughout the vasculature, showing the interaction of nanoparticles with the blood vessels and the ability to extravasate [170]. Along this transgenic line, other lines with fluorescent endothelial cells have been utilized for these types of assays, such as the Tg(kdrl:GFP)^la116tg^ line, which allowed the observation of the endocytosis of nanoparticles by endothelial cells and their behavior inside them [171]. 

Another important trait to evaluate is the time that nanoparticles can remain in the organism. This fact depends on the composition of the nanosystem and the response of the body’s immune system, such as macrophage uptake. Leveraging the zebrafish embryo transparency, microinjected fluorescent nanoparticles can be observed over time to evaluate their capacity to stay in the organism. An example that illustrates this usage is the study performed by Wang et al., involving nanosystems that can be used to carry anticancer drugs. In this study, FITC-labelled nanospheres were microinjected and evaluated during 72 h post-injection to study the biodistribution and their elimination progress of by the organism [172]. 

As the uptake of nanoparticles by macrophages decreases their half-life in circulation, one of the main objectives is testing the capacity of avoiding this nanoparticle uptake. To carry out this type of procedure, transgenic zebrafish reporter lines for fluorescently labelled macrophages, such as Tg(mpeg1:mCherry)^UMSF001^ and Tg(mpeg1:EGFP), have been used [173,174]. Making use of the Tg(mpeg1:mCherry)^UMSF001^ line, Evensen et al. studied the differences of anticancer nanoparticles with and without polyethylene glycol, observing a decrease in the uptake of the former by macrophages [173]. 

Though the evaluation of the behavior of microinjected nanoparticles is key, it is also important to study the effect of nanoparticles that are specifically developed for external treatments. In this field, Jia et al. developed a fluorescence probe, composed of cholesterol, poly(ethylene glycol)_2k_, and Cy5, for imaging zebrafish cell surfaces and demonstrated their utility for the assessment of nanoparticle toxicity in zebrafish upon observation of epidermal abnormalities related to damage [175].

#### 4.4.3. Anticancer Drug Delivery in Targeted Medicine

Zebrafish has turned into an anticancer nanomedicine platform to evaluate the efficacy of this treatment, since it allows the modeling of several types of cancer and different tumor stages, as explained in Section 3.1.

The transgenic Tg(FLK-1:EGFP) zebrafish line, which has green fluorescent endothelial cells, is one of the most common lines used to evaluate the antiangiogenesis capacity of drugs, including nanomedicines. The antiangiogenic effect of curcumin polymeric micelles was evaluated in this transgenic line, resulting in an effective inhibition of embryonic angiogenesis as well as tumor-derived angiogenesis owing to tumor cell xenotransplantation [170]. 

The microinjection of cancer cells allows assessing the capacity of nanoparticles to interact with xenotransplanted cells as well as their antitumoral efficacy [176,177,178,179]. A recent example is the work of Saraiva et al., who evaluated tumor reduction in xenografted zebrafish embryos treated with nanoemulsions comprising edelfosine, as a triple negative breast cancer treatment [180]. In a similar way, Moret et al. used zebrafish embryos that were the offspring of *Casper* mutants and fli1a:EGFP transgenic zebrafish to evaluate the efficacy of biodegradable poly(ethylene glycol)-poly(ε-caprolactone) nanoparticles, loaded with docetaxel, for epithelial cancer treatment, by measuring mass tumor reduction and antiangiogenic effect [181].

In addition, metastasis modelling in zebrafish embryos can be performed as a result of spreading across the circulation of xenotransplanted tumor cells. The microinjection or incubation of different types of antitumoral drugs allows the evaluation of their capacity to inhibit these metastatic processes [182,183].

## 5. Zebrafish as a Tool in Personalized Medicine

Chemotherapy treatments’ efficacy varies among different patients, and the results are not always successful [155]. For this reason, researchers are increasingly focusing on the development of personalized medicine strategies, such as the use of Patient Derived Xenografts (PDXs). PDXs allow the study of a particular tumor and its genome profile as well as its response to a specific treatment, owing to their capacity to maintain tumor heterogeneity. In recent years, zebrafish PDXs (zPDXs) have appeared as a new quick tool to evaluate anticancer treatments [155]. A role model was developed by Wu et al., who injected colon cancer cells from a patient into Tg(fli1:EGFP) transgenic zebrafish; the tumor–microenvironment interaction was observed, and a drug screening was carried, out allowing the selection of the most appropriate treatment for the patient based on the elimination of cancer cells [184]. This Tg(fli1:EGFP) zebrafish model, as well as the *casper* and *nacre* models, were used by Fior et al. to inject patient-derived colon cancer cells to screen different therapies. This experiment evidenced that xenografted zebrafish can be used as a fast screening platform to evaluate tumor evolution and relapse [155]. Furthermore, a *Casper* zebrafish model was used for the research of an effective treatment to inhibit leukemia cell proliferation through microinjection of patient-derived tumor cells [118] and was also used to study a chemotherapy drug combination in order to observe the reduction of tumor gastric mass [126]. In the same line of investigation, Usai et al. injected into wild-type zebrafish three different types of cancer cells from a patient, and different chemotherapy combinations were tested to determine the effective doses necessary to treat each cancer patient [114]. Another example of the use of *casper*, *nacre*, and Tg(fli1:EGFP) models is the work of Costa et al., in which a rectal cancer zPDX was generated and then treated with chemotherapy and radiotherapy to distinguish radiosensitive from radioresistant tumors [127].

Furthermore, in other studies, after zebrafish xenotransplantation of patient-derived cancer cells, PCR analysis was performed to analyze the efficiency of several drugs instead of measuring the reduction of tumors, achieving a new method to evaluate anticancer drug screenings [115]. 

In the field of nanomedicine, zebrafish also play a role as a model for personalized medicine in cancer. The work of Di Franco et al. is a clear example. Pancreatic cancer patient-derived xenografts were used to evaluate different therapies and to determine the best possible treatment for each patient. In this research, albumin nanoparticles loaded with Paclitaxel (nab-Paclitaxel), co-administrated with Gemcitabine, were one of the treatments tested. Overall, this study probes the potential of zebrafish for assessment of therapies based on nanotechnology by following a personalized approach [185].

## 6. Clinical Output 

The zebrafish as a preclinical disease model has proven to be key to inform about human disease mechanisms and therapy. Aside from the generation of powerful cancer models for the identification of therapeutic targets, this disease model plays an instrumental role in the era of precision medicine in oncology, allowing the tailoring of the treatments to the individual characteristics of each patient. Towards this end, recent efforts are being made to make use of the zebrafish as an “avatar” model for the xenotransplantation of cancer cells from individual patients (also known as zebrafish patient-derived xenografts or zPDX) and the subsequent studies of drug efficacy and response (Table 3). However, an important limitation in this regard has been the lack of criteria for the conversion of chemotherapy dosage from human to fish. This issue was recently addressed by Usai et al., who developed a formula to estimate equivalent doses (EDs) to be used on the fish [114]. The authors tested the ED for standard chemotherapy treatment in zebrafish xenotransplanted with cancer cell lines and confirmed their efficacy as determined in clinical studies [114]. Importantly, when tumor fragments derived from patients’ surgical specimens were engrafted in the fish, and these were treated with the ED of chemotherapy drugs, they found a good agreement with observations registered in common clinical practice. In line with this, similar evidence has been observed in other studies, such as the ones published by Fior et al., Costa et al., Rebelo de Almeida et al., and Di Franco et al., who showed a similar response of colorectal cancer and pancreatic cancer patients and zPDX to standard chemotherapy/chemoradiotherapy or targeted therapy for the treatment of these tumors [155,185,186,187]. In this regard, some preliminary indications have also been observed for zPDX derived from gastric cancer samples, although this needs further validation in a larger number of patient samples [184]. In addition, this strategy has been applied for non-solid tumors, such as multiple myeloma and B-cell precursor acute lymphoblastic leukemia, showing that zebrafish xenografts show similar responses to patients [188,189]. These proof-of-concept studies suggest that avatar/zPDX models can reproduce the individual response of each patient to treatment in just a few days, representing an important step forward towards the translation of this model into clinical practice as a predictive tool for the most effective treatment for an individual patient. Indeed, expanding on the initial work by Di Franco et al., a co-clinical trial is currently evaluating if zebrafish is able to predict the therapeutic regimen with the best efficacy for patients with pancreatic, gastrointestinal, or colorectal cancer undergoing chemotherapy in an estimated cohort size of 120 patients [190]. Currently, together with this co-clinical trial, another clinical study is listed on the website of Clinical Trials Gov, in which zebrafish is used as an avatar model [191]. In this trial (NCT01395628), which was already completed, zebrafish was evaluated as a recipient for primary human leukemia samples from 10 patients, to test the anti-proliferative or toxic effects of chemotherapeutics on them. It is worth noting that one of the main limitations of cancer models to predict patient drug responses is their limited complexity and capacity to recapitulate the intratumor heterogeneity, both genetic (clone selection) and cellular (stromal compartment). However, avatar xenotransplantation models generated from surgically resected specimens may preserve the actual complexity of the tumor, overcoming such an important limitation. Despite this, we cannot forget that these models may be limited by other factors, such as the successful engraftment in the fish of the tumor material and therefore the selection of specific tumor clones and the limited number of injected cells [192]. Therefore, despite the promising results of the zebrafish avatar/zPDX models for the prediction of patient treatment response, in order to achieve precision medicine through their use, larger clinical studies are needed to validate this strategy.

Aside from the potential use of the zebrafish as a predictive tool of patient drug response, this model system may also represent a valuable tool for the translation of compounds derived from zebrafish screens into the clinic as part of the personalized medicine approach. Two decades ago, zebrafish was mainly used for the development of chemical phenotypic screens, meaning the screening for compounds with therapeutic properties over a specific phenotype or disease. In the last ten years or so, zebrafish models are being used following a “from bench to bedside” approach to identify the best treatment plan for an individual patient. In this sense, the screening of compounds in the zebrafish for the development of selective therapies has experienced a relative advance in recent years, with some compounds already being tested in clinical trials, or close to it, for the treatment of various diseases, including cancer [193]. This experimental approach has been pioneered by the Group of Leonard I Zon at Harvard, who has contributed to the repurposing (or reprofiling) of four compounds, two of them as anti-cancer agents and one for graft-versus-host disease in hematologic malignancies. The first example is ProHema, a derivative of prostaglandin E2 shown to increase the generation of blood stem cells, which was repurposed for its use in blood stem cell transplantation [194]. ProHema has been tested in four phase I or II clinical trials, in three of which it was evaluated for its efficacy in hematologic malignancies (NCT00890500, NCT01627314, and NCT02354417). This compound was later incorporated into a cellular immunotherapy (ProTmune; Fate Therapeutics) used for the prevention of graft-versus-host disease and is currently being evaluated in an ongoing phase II stage clinical trial in hematologic malignancies (NCT02743351). The second compound is the antirheumatic drug Leflunomide, an inhibitor of the dihydroorotate dehydrogenase, which was identified in a zebrafish screening as a drug with the potential to interfere with the growth of melanoma [195]. The drug was included in a phase I clinical trial to test its efficacy in combination with a BRAF inhibitor (NCT01611675), but it was later canceled due to adverse events. The third compound is the all-trans retinoic acid (ATRA), in this occasion identified using a pluripotent zebrafish blastomere culture system, which was shown to suppress the transcription factor c-myb, a driver of adenoid cystic carcinoma [196]. These findings led to the initiation of a phase II clinical trial (*n* = 18) evaluating the safety and effectiveness of ATRA in treating adenoid cystic carcinoma, completed last year (NCT03999684), in which patient response to ATRA was not observed for the tested dose and schedule [197]. Currently, a second phase II trial (*n* = 30) is underway, testing the compound in patients with recurrent metastatic adenoid cystic carcinoma of the head and neck (NCT04433169). Another good example of this research approach is Rosuvastatin, used for the treatment of hypercholesterolemia in cardiovascular disorders and repurposed as an antiangiogenic drug after a genetic screen in zebrafish [198]. Rosuvastatin was shown to inhibit the growth of prostate cancer cells and was later included in a phase II clinical trial to evaluate the improvement in the response to its combination with standard chemotherapy in rectal cancer (NCT02569645, still recruiting an estimate of 48 patients). Moreover, zebrafish also allows for the testing of combinatory treatments. A very recently developed immune-deficient adult zebrafish model (*prkdc*^−/−^, *il2rga*^−/−^) by David Langenau at the Massachusetts General Hospital Research Institute has been used for the identification of a possible treatment for rhabdomyosarcoma [95]. The researchers made use of this model to prove the efficacy of the combination of a poly ADP ribose polymerase (PARP) inhibitor plus temozolomide chemotherapy, both drugs approved for use in the clinic, in eliminating engrafted rhabdomyosarcoma cells, as opposed to single drug treatment, an effect that was confirmed in a mouse xenograft model [95]. This combinatorial treatment is being investigated in a phase I study for Ewings sarcoma or Rhabdomyosarcoma in an estimated cohort of 93 patients (NCT01858168).

In addition to the above examples, some other clinically approved compounds, repurposed through zebrafish screening, have not reached the clinical testing stage. Perphenazine (PPZ), a drug approved for psychosis therapy, was found to be effective against T-cell acute lymphoblastic leukemia (T-ALL) in a combined screening for small molecules with toxic effect in MYC-overexpressing thymocytes in zebrafish and T-ALL cells [199]. More recently, a potential use of PPZ for the treatment of endometrial cancer has been suggested based on in vitro and mouse experimental data, expanding the potential use of this compound for solid tumors [200]. A zebrafish genetic model of β-catenin driven hepatocellular carcinoma (HCC) allowed the identification of two antidepressants, amitriptyline and paroxetine, as suppressors of liver growth [201]. Further experiments developed in this study have shown that paroxetine was also able to decrease tumor burden in a mouse HCC model [201]. Another successful reprofiling example was shown by Fernandez del Ama et al., who, using an oncogenic-RAS-driven zebrafish melanoma model, observed that the mTOR inhibitor rapamycin, as well as the compounds disulfiram and tanshinone, synergized with inhibitors of the MEK and PI3K/mTOR signaling pathways to inhibit melanoma development [202].

In summary, we can clearly see the huge role of zebrafish as a cancer model in the development of pre-clinical studies for the identification of compounds with antitumoral properties. However, the identification of anticancer compounds is not enough to make an impact on cancer patient care, as most do not reach the clinical testing, and more direct approaches are needed. In this sense, the field is drifting towards the development and use of zebrafish avatar models for the testing of patient drug sensitivity, since each individual patient may present a different response based on the unique genetic alterations that his/her tumor harbors. Particularly, future work should be aimed at testing the predictive value of zebrafish avatars on a reduced number of therapeutic options (targeted therapies and immunotherapies) but in larger patient cohorts, in order to achieve a truly personalized treatment. The xenograft approach is supported by multiple studies that have validated the development of zebrafish xenografts from patient-derived material [203], and, as discussed before, more studies are coming out showing the true potential of the zebrafish avatars for this purpose, predicting patient responses [115,139,155,156,184,185,186,187,189].

## 7. Conclusions

The zebrafish is a powerful model for studies of various cancer treatments, including new therapies, such as those based on nanomedicine. Its versatility, which allows it to be used not only in embryonic stages but also as adult individuals, together with the enormous variety of transgenic lines available, are fundamental characteristics of this model. However, more research efforts should be directed toward the development of standardized protocols for tumor cell xenotransplantation and drug effectiveness analysis, as well as toward optimizing the routes of administration in order to translate the results to higher models and more patients. Moreover, since most of the research is performed in larvae, long-term drug exposure and the assessment of response lack translatability to patients, and for that other animal models are needed.

Furthermore, the use of zebrafish as an intermediate step between cell culture studies and higher models, such as mice and rats, reduces the number of seconds needed to perform the experiments, thus implementing the 3R (replacement, reduction, and refinement) principle of animal welfare.

## Figures and Tables

**Table 1:** Benefits and drawbacks of using zebrafish for modeling human diseases in comparison with other animal models.

Advantages	Disadvantages
Simple anatomy	Some mammalian organs are missing
External fertilization	Optimal temperature at 28 °C, compromising human cell viability
Embryo and larvae optical transparency	Lack of sexual chromosomes
Rapid development and sexual maturation	Pooling individuals prevent the observation of interindividual differences
High fertility rates	Mice genetic homology is higher
Large number of individuals and statistical power	Low amount of certain tissues for biological assays
Robust embryos	Genetic duplication
High homology in human disease-related genes	Protocol variability, limiting the comparison among studies
Late activation of the adaptive immune system	Need of mammal models for further preclinical studies
Cost-effective and easy maintenance	Low antibodies availability for molecular assays
Easy genetic manipulation	
Low number of cells for xenograft assays	
Availability of reporter lines	
Many existing zebrafish resources and repositories	

**Table 3:** Studies involving the use of zebrafish PDXs for drug efficacy and response.

Tumor Type	Patients (*n*)	Aim	Outcome	Ref.
Pancreatic (PC), colorectal (CRC), and gastric cancer (GC)	*n* = 24 (12 PC, 8 CRC, and 4 GC patients)	Xenograft establishment (*n* = 6) Response to chemotherapy options (according to the cancer type) evaluated as partial response (PR) and complete response (CR)	Xenografted tumor tissue can engraft and survive in the zebrafish (100%). Response to chemotherapy: ▪ PC: PR to GEM/nab-P (58.33 %), GEM (50%), GEMOX (50%), and FOLFOXIRI (33.33 %). No CR was observed. ▪ CRC: PR to FOLFOX, FOLFIRI and FOLFOXIRI (62.5%), and to 5-FU (37.5%). CR to FOLFIRI (12.5%). ▪ GC: PR to FOLFIRI (100%), FOLFOX, FLOT and ECF (25%). CR to FOLFIRI (25%).	[114]
Colorectal cancer (CRC)	*n* = 11	Xenograft establishment (*n* = 5) Sensitivity to standard chemotherapy and targeted therapy	Cell engrafted in 5/5 cases (100%), with different success rates based on the percentage of fish showing engraftment (from 47 to 89%). Zebrafish xenograft response to FOLFOX anticipated patient relapse/no relapse within 3 m to 6 m in 4/5 patients (80%). Lack of response to Cetuximab was associated with mutations highly linked to Cetuximab resistance.	[155]
Gastric cancer (GC)	*n* = 14	Xenograft establishment Assess the efficacy of anti-GC agents: 5-FU, docetaxel, and apatinib (*n* = 4)	Successful transplantation in 9/14 patient samples (64.2 %). Zebrafish xenografts subjected to 5-FU and apatinib showed different degrees of sensitivity.	[184]
Pancreatic ductal adenocarcinoma (PDAC)	*n* = 15	Xenograft establishment Evaluation of response to chemotherapy	Establishment of PDAC xenografts in 15/15 cases (100%). Significant reduction in tumor area observed in 6/15 cases (40%) for at least one chemotherapy scheme (FOLFOXIRI, GEMOX, Gem/nab-P, and GEM.	[190]
Breast (BC) and colorectal cancer (CRC)	*n* = 6 (3 BC and 3 CRC patients)	Response to the anti-VEGF therapy bevacizumab Comparison of patient’s response with matching avatars (*n* = 2)	Zebrafish avatars can reflect both pro- and anti-metastatic effects of bevacizumab. Resistance to bevacizumab of zebrafish avatar correlation with the clinical resistance and disease progression of the matched patients.	[187]
Multiple myeloma (MM)	*n* = 6	Xenograft establishment (perivitelline space) Evaluate drug response in newly diagnosed (*n* = 2) and relapsed/refractory patients (*n* = 4)	Efficiency of MM primary cell engraftment of around 80%. Zebrafish xenograft responses to bortezomib and lenalidomide recapitulated patient responses in all 6 cases.	[189]
B-cell precursor acute lymphoblastic leukemia (BCP-ALL)	*n* = 15	Xenograft establishment (pericardium) Response of BCP-ALL cell lines to venetoclax (*n* = 7)	BCP-ALL were successfully expanded in 9/15 embryos (60%). Xenografts produced varied responses to venetoclax, mirroring in two cases the refractory response to venetoclax of the matching patients.	[188]

Abbreviations: ECF: 5-Fluorouracil + Cisplatin + Epirubicin; FOLFIRI: 5-Fluorouracil + Lederfolin + Irinotecan; FOLFOX: 5-Fluorouracil + Lederfolin + Oxaliplatin; FOLFOXIRI: 5-Fluorouracil + Folinic acid + Oxaliplatin + Irinotecan; FLOT: 5-Fluorouracil + Lederfolin + Oxaliplatin + Docetaxel; GEM: Gemcitabine; GEMOX: Gemcitabine + Oxaliplatin; GEM/nab-P: Gemcitabine + nab-Paclitaxel; 5-FU: 5-Fluorouracil.

## Abbreviations

AgNPs	Silver nanoparticles
ATRA	All-trans retinoic acid
AuNPs	Golden nanoparticles
BC	Breast cancer
BPC-ALL	B-cell precursor acute lymphoblastic leukemia
CAR-T cell	Chimeric antigen receptor T cell
CR	Complete response
CRC	Colorectal cancer
NSCLC	Non-small-cell lung cancer
CTLA-4	Cytotoxic T-lymphocyte-associated antigen-4
DEN	Diethylnitrosamine
DMBA	Dimethylbenzanthracene
Dpf	Days post-fertilization
ED	Equivalent doses
EGFR	Epidermal growth factor receptor
EMA	European Medicines Agency
ENU	Ethylnitrosourea
FDA	Food and Drug Administration
GBM	Glioblastoma
GC	Gastric cancer
GCT	Germ cell tumor
HCC	Hepatocellular carcinoma
Hpf	Hours post-fertilization
ICIs	Immune checkpoint inhibitors
ISVs	Intersegmental vessels
MET	Mesenchymal-epithelial transition
MM	Multiple myeloma
MMDOX	Doxorubicin-loaded mixed micelles
MNNG	N1-nitro-N-nitrosoguanidine
MO	Morpholinos
MPNSTs	Malignant peripheral nerve sheath tumors
MSNs-FA	Mesoporous silica nanoparticles coated with folic acid
NDMA	N-nitrosodimethylamine
NF1	Neurofibromin 1
NK	Natural killer cells
PARP	Poly ADP ribose polymerase
PC	Pancreatic cancer
PD-1	Programmed cell death-1
PDAC	Pancreatic ductal adenocarcinoma
PD-L1	Programmed cell death ligand-1
PDX	Patient Derived Xenografts
PlexA1	Plexin-A1
PPZ	Perphenazine
PR	Partial response
pVHL	Von Hippel-Lindau protein
shRNA	Short hairpin RNA
TALENs	Transcription Activator-Like Effector Nucleases
T-ALL	T-cell acute lymphoblastic leukemia
TILLING	Targeted Induced Local Lesions in Genomes
VEGF	Vascular endothelial growth factor
Wpf	Weeks post-fertilization
ZFNs	Zinc Finger Nucleases
zPDXs	zebrafish patient-derived xenografts
